# The Psychology of Food Cravings: the Role of Food Deprivation

**DOI:** 10.1007/s13668-020-00326-0

**Published:** 2020-06-23

**Authors:** Adrian Meule

**Affiliations:** 1 Department of Psychiatry and Psychotherapy, University Hospital, LMU Munich, Munich, Germany; 2 Schoen Clinic Roseneck, Am Roseneck 6, 83209 Prien am Chiemsee, Germany

**Keywords:** Food craving, Dieting, Food deprivation, Caloric restriction, Pavlovian conditioning, Extinction

## Abstract

**Purpose of Review:**

Dieting is often blamed for causing food cravings. Such diet-induced cravings may be mediated by physiological (e.g., nutritional deprivation) or psychological (e.g., ironic effects of food thought suppression) mechanisms. However, this notion is often based on cross-sectional findings and, thus, the causal role of food deprivation on food cravings is unclear.

**Recent Findings:**

Experimental studies suggest that a short-term, selective food deprivation seems to indeed increase cravings for the avoided foods. However, experimental studies also show that food craving can be understood as a conditioned response that, therefore, can also be unlearned. This is supported by intervention studies which indicate that long-term energy restriction results in a reduction of food cravings in overweight adults.

**Summary:**

Dieting’s bad reputation for increasing food cravings is only partially true as the relationship between food restriction and craving is more complex. While short-term, selective food deprivation may indeed increase food cravings, long-term energy restriction seems to decrease food cravings, suggesting that food deprivation can also facilitate extinction of conditioned food craving responses.

## Introduction

A food craving is an intense desire to eat a particular type of food [1]. Humans typically crave energy-dense foods: chocolate and other chocolate-containing foods are the most frequently craved foods, followed by other high-caloric sweet and savory foods [2–6]. Cultural differences have been found, for example, with rice being the most frequently craved food in Japan [7]. Among low-caloric foods, cravings for fruits are also reported quite commonly [3, 5, 8]. Food cravings typically occur in the late afternoon and evening [9]. Interestingly, only the desire to eat high-calorie foods increases throughout the day, while craving for fruits decreases [8].

Hunger refers to the absence of fullness, that is, feelings of hunger are brought about by an empty stomach [10]. Food craving can be differentiated from feelings of hunger through its specificity and intensity. That is, while a food craving can usually only be satisfied by consumption of a particular food, hunger can be alleviated by consumption of any type of food [11]. Moreover, while hunger and food craving often can co-occur, being hungry is not a prerequisite for experiencing a food craving. In a laboratory study on chocolate craving, for example, current chocolate craving intensity was positively correlated with current hunger, but unrelated to the length of food deprivation. Moreover, only current chocolate craving intensity—but not current hunger—related to higher salivary flow during a chocolate exposure and to higher chocolate consumption [12].

The experience of a food craving is multidimensional. Physiologically, it is associated with several processes that prepare the body for ingestion and motivates food seeking and consumption such as increased salivary flow [12, 13] and activation of reward-related brain areas such as the striatum [14–16]. It also includes cognitive (i.e., thinking abou the food) and emotional (e.g., desire to eat or changes in mood) components. Finally, it often also includes a behavioral component of seeking and consuming the food. Yet, while experiencing a food craving often leads to consumption of the craved food, the craving–consumption relationship also depends on interindividual differences and situational factors [3, 17].

## Theories of Food Craving

It seems obvious to assume that the emergence of a food craving might be driven by some nutrient deficiency. However, evidence for this is relatively poor. For example, when participants had to consume a nutritionally balanced, yet monotonous, liquid diet, they reported more food cravings than during a baseline period [18], and food craving could be induced by imagining their favorite food although participants were sated [16]. During pregnancy—a time during which the body needs more energy and certain nutrients than usual—it seems that the types of craved foods do not differ from usually craved foods [19, 20], and even if women crave unusual, potentially harmful, foods or other substances, it seems that this is rather driven by social factors than by physiological needs [21]. Similar interpretations have been derived from perimenstrual (chocolate) cravings which, for example, do not disappear after menopause, making hormonal mechanisms unlikely [11].

Hence, although simple associations between nutrient deficiency and food cravings seem compelling, they appear to account for a small fraction of food cravings at most. Instead, several psychological explanations for why and how food cravings emerge have been developed. Prominent models are based on (Pavlovian) conditioning [22]. Here, a cue or context that has been repeatedly paired with food intake can itself elicit a conditioned response (e.g., food craving) that promotes food intake. Theoretically, any cue can become a conditioned stimulus that elicits food craving and, indeed, appetitive conditioning studies in the laboratory have shown that neutral stimuli such as geometric figures or objects can increase eating desires when they have previously been associated with food intake [23•]. In real life, relevant cues are typically internal states such as hunger [24], external states or contexts such as time of day [25], and many more [23•].

There are also several cognitive models which, however, often include reference to conditioning processes but additionally assume higher-order cognitive processes that play a role in the craving response (see [26•] for an overview of different theories). For example, an important part of the Elaborated Intrusion Theory of Desire [27–29] is that while cues can unconsciously trigger intrusive images or thoughts, these thoughts are then consciously elaborated by retrieving cognitive associations and creating mental imagery of the target. Others emphasize the ambivalent nature of food cravings, which are often marked by both approach and avoidance inclinations that can be partially attributed to socio-cultural norms [20, 30, 31].

## Dieting and Food Craving

There has been a considerable interest in the question of whether food restriction induces food cravings. Research on this topic was started off as early as the 1970s and heavily influenced by a now classic study by Herman and Mack [32]. They found that so-called restrained eaters showed a disinhibited eating behavior (higher ice cream consumption) after consumption of a preload (milkshake), whereas so-called unrestrained (“normal”) eaters decreased their ice cream consumption after consumption of a milkshake. This effect was later assumed to stem from the fact that restrained eaters had violated their dietary rules (exceeded their dietary boundary) by consumption of the milkshake, which led to a “what-the-hell-effect” [33]. Later research showed that there are many other factors (e.g., distraction, emotions) that can disinhibit food intake in restrained eaters [34, 35].

Self-report measures of restrained eating are positively correlated with self-report measures of food craving. That is, restrained eaters experience more intense and more frequent food cravings than unrestrained eaters [36]. Yet, it has also been demonstrated that measures of restrained eating do not relate to actual caloric restriction [37–39]. Thus, it appears that restrained eaters should rather be regarded as eating- and weight-concerned persons who may or may not be actually dieting [40, 41].

Although restrained eating and dieting are not synonymous, studies that differentiated more clearly between current dieters and non-dieters seem to show similar results to the restrained eating literature. In a study by Massey and Hill [42], for example, food cravings were measured with daily diaries across 7 days and it was found that current dieters reported more food cravings than non-dieters. However, a causal influence of dieting on the likelihood of experiencing food cravings can still not be inferred from such studies as dieters may have a higher predisposition for experiencing cravings in the first place. In other words, the susceptibility for experiencing cravings and giving in to them may lead to weight gain and subsequent dieting attempts (and not the other way around).

## Effects of a Selective Food Deprivation

As cross-sectional research based on self-reported restrained eating or dieting cannot clearly answer the question of whether food restriction causes food cravings, experimental studies have been conducted. One type of such studies investigated a selective food deprivation during which participants were instructed to refrain from eating certain types of food. This type of experimental manipulation is sometimes also called a “hedonic deprivation” [43] as only specific foods are avoided, while consumption of all other foods are unrestricted and, thus, total energy consumption is assumed to be unaffected.

Table 1 lists the specifications and results of such studies. Some studies included a deprivation of chocolate-containing foods but single studies on other food types are also available. The deprivation periods ranged between 1 day and 14 days, and all studies investigated university students. Almost all studies found that the deprivation increased cravings for the avoided foods. An exception is the study by Polivy and colleagues [49] who did not find deprivation-induced effects on craving. However, they found that chocolate-deprived restrained eaters ate the largest amount of chocolate in a laboratory taste test. These results are partially in line with the findings by Richard and colleagues [50] who found deprivation effects only in trait chocolate cravers. Thus, it seems that deprivation-induced increases in food craving can primarily be found in a subgroup of susceptible individuals such as restrained eaters or trait food cravers.

**Table 1:** Experimental studies on the effects of selective food deprivation on food craving in humans

Reference	*n*	Participants	Avoided foods	Length of deprivation period	Findings
Beauchamp et al. [44]	10	University students (40% women)	Sodium-rich foods	10 days	Deprivation-induced increase in desire to eat salty foods
Blechert et al. [45]	29	University students (100% women)	Chocolate-containing foods	7 days	Deprivation-induced increase in desire to eat chocolate-containing foods
Coelho et al. [46]	77	University students (100% women)	Carbohydrate-rich or protein-rich foods	3 days	Deprivation-induced increase in carbohydrate-rich food craving frequency or protein-rich food craving frequency
Komatsu et al. [47] (Study 1)	51	University students (84% women)	Rice	1 day or 3 days	Deprivation-induced increase in rice craving frequency
Komatsu et al. [47] (Study 2)	28	University students (57% women)	Bread	3 days	Deprivation-induced increase in bread craving frequency
Moreno et al. [48]	58	University students (100% women)	Chocolate-containing foods	14 days	Deprivation-induced increase in desire to eat chocolate-containing foods
Polivy et al. [49]	103	University students (100% women)	Chocolate-containing or vanilla-containing foods	7 days	No effects of deprivation on food cravings
Richard et al. [50]	60	University students (75% women)	Chocolate-containing foods	14 days	Deprivation-induced increase in desire to eat chocolate-containing foods in high trait chocolate cravers (but not in low trait chocolate cravers)

In sum, these studies support psychological mechanisms in the emergence of food cravings as the selective food deprivation instructions are unlikely to have created a nutrient deficiency. An exception may be the study by Beauchamp and colleagues [44] during which a very low sodium diet was complemented by the administration of diuretics to achieve sodium depletion. Because of this, however, this sodium depleted state seems not to be representative for the average individual who exhibits dietary restraint or engages in weight-loss dieting. As none of these studies imposed a restriction on any foods other than the avoided foods or on total caloric intake, it seems that perceived deprivation—a feeling of not eating what or as much as one would like, despite being in energy balance [51]—plays a larger part in generating food cravings than actual nutrient deficiencies.

## Effects of Energy-Restricting Diets

Another type of studies that is relevant to consider when examining whether food restriction causes food cravings are weight-loss interventions. As opposed to selective food deprivation studies, weight-loss interventions aim to create an energy deficit, which is primarily achieved by a reduction of energy intake (although they may also involve physical activity to increase energy expenditure). Importantly, results from these studies are opposite to the findings from selective food deprivation studies. A meta-analysis by Kahathuduwa and colleagues [52•] which investigated eight studies in which caloric restriction was used as part of a weight-loss intervention found that food cravings actually decrease from pre- to post-intervention. Another systematic review that examined similar studies came to the same conclusion [53].

Table 2 lists the most recent studies between 2017 and 2020 that were not included in the meta-analysis by Kahathuduwa and colleagues [52•]. Intervention periods ranged between 4 weeks and 2 years and all studies investigated overweight or obese adults. An exception is the study by Dorling and colleagues [58] which also included normal-weight participants. This is also the study for which results were not entirely clear as the changes in food cravings were moderated by sex. Yet, findings from all other studies are in line with the meta-analytical results by Kahathuduwa and colleagues [52•] as food cravings decreased during the caloric restriction period. These decreases seem to occur primarily during the first weeks of caloric restriction and do not seem to rebound at later follow-up measurements [60, 62, 63].

**Table 2:** Intervention studies on the effects of an energy-restricting diet on food craving in humans

Reference	*n*	Participants	Intervention	Length of intervention period	Findings
Anguah et al. [54]	19	Overweight adults (47% women)	Low-carbohydrate diet (1500 kcal/day)	4 weeks	Reductions in total cravings (i.e., for different kind of foods) from pre- to post-intervention
Apolzan et al. [55•]	367	Overweight adults (56% women)	Four different diets (750 kcal/day deficit from baseline energy requirements)	2 years	Reductions in total cravings (i.e., for different kind of foods) after 6 months
Castro et al. [56]	20	Obese adults (60% women)	Very low-calorie ketogenic diet (600–800 kcal/day during the first 60–90 days)	4 months	Reductions in total cravings (i.e., for different kind of foods) from pre- to post-intervention
Chao et al. [57]	178	Obese adults (88% women)	Group behavioral weight loss program with a structured meal replacement (1000–1200 kcal/day between weeks 2 and 12)	14 weeks	Reductions in total cravings (i.e., for different kind of foods) from pre- to post-intervention
Dorling et al. [58]	218	Normal-weight and overweight adults (70% women)	Energy restriction (25% decreased energy intake from energy requirements)	2 years	Men (but not women) in the caloric restriction group showed reduced carbohydrate and fat cravings at 24 months as compared to the ad libitum eating group
Drapeau et al. [59]	100	Overweight adults (71% women)	Energy restriction of 500–700 kcal/day	12–15 weeks	Reductions in total cravings (i.e., for different kind of foods) from pre- to post-intervention
Morin et al. [60]	60	Overweight adults (100% women)	Energy restriction with or without cognitive dietary restraint induction (15% decreased energy intake from energy requirements)	4 weeks	Reductions in total cravings (i.e., for different kind of foods) from pre- to post-intervention and at 16 weeks follow-up
Smithson and Hill [61]	1224	Overweight adults (97% women)	Group-based weight management program (Slimming World)	7 Weeks	Reductions in total cravings (i.e., for different kind of foods) from pre- to post-intervention
Watson et al. [62]	61	Overweight adults with Type-2-diabetes (46% women)	High-protein or high-carbohydrate diet (1500 kcal/day)	12 weeks	Reductions in total cravings (i.e., for different kind of foods) from pre- to post-intervention and at 24 weeks follow-up

Only recent studies that are not included in the meta-analysis by Kahathuduwa et al. [52•] are described

In sum, results from caloric restriction studies again speak against the notion that nutrient deficiencies or an energy deficit cause food cravings and instead favor psychological explanations. For example, decreases in food cravings during caloric restriction may be due to extinction of previously acquired conditioned responses [23•, 26•]. In line with this is that a reduced frequency (but not the amount) of consuming craved foods related to reductions in cravings for these foods in the study by Apolzan and colleagues [55•]. That is, not eating certain foods over a period of at least several weeks may decouple learned associations (e.g., between evening time and chocolate consumption) so that certain cues (e.g., evening time) do no longer trigger a conditioned response (e.g., craving for chocolate).

## Conclusions

At first glance, it appears that the literature on the effects of food restriction on food cravings produced ambiguous findings. While selective food deprivation seems to increase food cravings, caloric restriction seems to decrease them. However, these contradictory findings may be explained by several methodological differences between these studies. First, selective food deprivation studies have exclusively been conducted in university students, most of them normal-weight women (Table 1). In contrast, caloric restriction studies have almost exclusively been conducted in overweight persons (Table 2). Thus, it cannot be excluded whether different types of food restriction have different effects on craving as a function of sample characteristics such as body weight. Second, selective food deprivation studies included deprivation periods of few days up to 2 weeks, while caloric restriction studies included periods of several weeks and months. Thus, it may be that avoiding certain foods may increase cravings in the first few days but that they may have decreased if the selective food deprivation studies would have been conducted over longer periods of time. This interpretation is in line with the fact that extinction learning takes longer than acquisition [23•].

In conclusion, a nutrient deficiency or an energy deficit brought about by food restriction can rarely explain the emergence of a food craving (although this may be true in some rare cases [64, 65] or if food intake is completely terminated [66, 67]). Instead, food craving can rather be understood as a conditioned response that emerges because internal or external cues have been previously associated with intake of certain foods. Restrained eaters, dieters, and study volunteers that are instructed to refrain from eating certain foods report more cravings for these foods over short periods of several days. However, weight-loss studies in overweight individuals consistently show that caloric restriction leads to decreases in food cravings, which may be due to extinction processes when certain foods are avoided and substituted with healthier alternatives. Thus, the wide-held notion that dieting inevitably leads to food cravings is strongly oversimplified as the relationship between food restriction and food craving is more complex.

## References

[1] Weingarten HP, Elston D (1990). The phenomenology of food cravings. Appetite..

[2] Weingarten HP, Elston D (1991). Food cravings in a college population. Appetite..

[3] Richard A, Meule A, Reichenberger J, Blechert J (2017). Food cravings in everyday life: an EMA study on snack-related thoughts, cravings, and consumption. Appetite..

[4] Osman JL, Sobal J (2006). Chocolate cravings in American and Spanish individuals: biological and cultural influences. Appetite..

[5] Hill AJ, Heaton-Brown L (1994). The experience of food craving: a prospective investigation in healthy women. J Psychosom Res.

[6] Zellner DA, Garriga-Trillo A, Rohm E, Centeno S, Parker S (1999). Food liking and craving: a cross-cultural approach. Appetite..

[7] Komatsu S (2008). Rice and sushi cravings: a preliminary study of food craving among Japanese females. Appetite..

[8] Reichenberger J, Richard A, Smyth JM, Fischer D, Pollatos O, Blechert J (2018). It's craving time: time of day effects on momentary hunger and food craving in daily life. Nutrition..

[9] Pelchat ML (1997). Food cravings in young and elderly adults. Appetite..

[10] Rogers PJ, Brunstrom JM (2016). Appetite and energy balancing. Physiol Behav.

[11] Hormes JM, Hollins-Martin C, van den Akker O, Martin C, Preedy VR (2014). Perimenstrual chocolate craving: from pharmacology and physiology to cognition and culture. Handbook of diet and nutrition in the menstrual cycle, periconception and fertility.

[12] Meule A, Hormes JM (2015). Chocolate versions of the food cravings questionnaires. Associations with chocolate exposure-induced salivary flow and ad libitum chocolate consumption. Appetite..

[13] Nederkoorn C, Smulders F, Jansen A (2000). Cephalic phase responses, craving and food intake in normal subjects. Appetite..

[14] Contreras-Rodríguez O, Martín-Pérez C, Vilar-López R, Verdejo-Garcia A (2017). Ventral and dorsal striatum networks in obesity: link to food craving and weight gain. Biol Psychiatry.

[15] Miedl SF, Blechert J, Meule A, Richard A, Wilhelm FH (2018). Suppressing images of desire: neural correlates of chocolate-related thoughts in high and low trait chocolate cravers. Appetite..

[16] Pelchat ML, Johnson A, Chan R, Valdez J, Ragland JD (2004). Images of desire: food-craving activation during fMRI. NeuroImage..

[17] Richard A, Meule A, Blechert J (2019). Implicit evaluation of chocolate and motivational need states interact in predicting chocolate intake in everyday life. Eat Behav.

[18] Pelchat ML, Schaefer S (2000). Dietary monotony and food cravings in young and elderly adults. Physiol Behav.

[19] Hill AJ, Cairnduff V, McCance DR (2016). Nutritional and clinical associations of food cravings in pregnancy. J Hum Nutr Diet.

[20] Orloff NC, Hormes JM (2014). Pickles and ice cream! Food cravings in pregnancy: hypotheses, preliminary evidence, and directions for future research. Front Psychol.

[21] Placek C (2017). A test of four evolutionary hypotheses of pregnancy food cravings: evidence for the social bargaining model. R Soc Open Sci.

[22] Jansen A (1998). A learning model of binge eating: cue reactivity and cue exposure. Behav Res Ther.

[23] van den Akker K, Schyns G, Jansen A (2018). Learned overeating: applying principles of pavlovian conditioning to explain and treat overeating. Curr Addict Rep.

[24] Gibson EL, Desmond E (1999). Chocolate craving and hunger state: implications for the acquisition and expression of appetite and food choice. Appetite..

[25] van den Akker K, Havermans RC, Jansen A (2017). Appetitive conditioning to specific times of day. Appetite..

[26] Tapper K (2018). Mindfulness and craving: effects and mechanisms. Clin Psychol Rev.

[27] Kavanagh DJ, Andrade J, May J (2005). Imaginary relish and exquisite torture: the elaborated intrusion theory of desire. Psychol Rev.

[28] May J, Andrade J, Kavanagh DJ, Hetherington M (2012). Elaborated intrusion theory: a cognitive-emotional theory of food craving. Curr Obes Rep.

[29] May J, Kavanagh DJ, Andrade J (2015). The elaborated intrusion theory of desire: a 10-year retrospective and implications for addiction treatments. Addict Behav.

[30] Cartwright F, Stritzke WGK (2008). A multidimensional ambivalence model of chocolate craving: construct validity and associations with chocolate consumption and disordered eating. Eat Behav.

[31] Hormes JM, Niemiec MA (2017). Does culture create craving? Evidence from the case of menstrual chocolate craving. PLoS One.

[32] Herman CP, Mack D (1975). Restrained and unrestrained eating. J Pers.

[33] Herman CP, Polivy J (1983). A boundary model for the regulation of eating. Psychiatr Ann.

[34] Evers C, Dingemans A, Junghans AF, Boevé A (2018). Feeling bad or feeling good, does emotion affect your consumption of food? A meta-analysis of the experimental evidence. Neurosci Biobehav Rev.

[35] Stroebe W, van Koningsbruggen GM, Papies EK, Aarts H (2013). Why most dieters fail but some succeed: a goal conflict model of eating behavior. Psychol Rev.

[36] Meule A, Lutz A, Vögele C, Kübler A (2012). Food cravings discriminate differentially between successful and unsuccessful dieters and non-dieters. Validation of the food cravings questionnaires in German. Appetite..

[37] Stice E, Cooper JA, Schoeller DA, Tappe K, Lowe MR (2007). Are dietary restraint scales valid measures of moderate- to long-term dietary restriction? Objective biological and behavioral data suggest not. Psychol Assess.

[38] Stice E, Fisher M, Lowe MR (2004). Are dietary restraint scales valid measures of acute dietary restriction? Unobtrusive observational data suggest not. Psychol Assess.

[39] Stice E, Sysko R, Roberto CA, Allison S (2010). Are dietary restraint scales valid measures of dietary restriction? Additional objective behavioral and biological data suggest not. Appetite..

[40] Lowe MR (1995). Restrained eating and dieting: replication of their divergent effects on eating regulation. Appetite..

[41] Lowe MR, Timko CA (2004). What a difference a diet makes: towards an understanding of differences between restrained dieters and restrained nondieters. Eat Behav.

[42] Massey A, Hill AJ (2012). Dieting and food craving. A descriptive, quasi-prospective study. Appetite..

[43] Conason A, Avena NM (2015). The influence of dieting (hedonic deprivation) on food intake, how it can promote hedonic overeating, and mindful-eating interventions. Hedonic eating.

[44] Beauchamp GK, Bertino M, Burke D, Engelman K (1990). Experimental sodium depletion and salt taste in normal human volunteers. Am J Clin Nutr.

[45] Blechert J, Naumann E, Schmitz J, Herbert BM, Tuschen-Caffier B (2014). Startling sweet temptations: hedonic chocolate deprivation modulates experience, eating behavior, and eyeblink startle. PLoS One.

[46] Coelho JS, Polivy J, Herman CP (2006). Selective carbohydrate or protein restriction: effects on subsequent food intake and cravings. Appetite..

[47] Komatsu S, Kyutoku Y, Dan I, Aoyama K (2015). Rice deprivation affects rice cravings in Japanese people. Food Qual Prefer.

[48] Moreno-Dominguez S, Rodríguez-Ruiz S, Martín M, Warren CS (2012). Experimental effects of chocolate deprivation on cravings, mood, and consumption in high and low chocolate-cravers. Appetite..

[49] Polivy J, Coleman J, Herman CP (2005). The effect of deprivation on food cravings and eating behavior in restrained and unrestrained eaters. Int J Eat Disord.

[50] Richard A, Meule A, Friese M, Blechert J (2017). Effects of chocolate deprivation on implicit and explicit evaluation of chocolate in high and low trait chocolate cravers. Front Psychol.

[51] Markowitz JT, Butryn ML, Lowe MR (2008). Perceived deprivation, restrained eating and susceptibility to weight gain. Appetite..

[52] Kahathuduwa CN, Binks M, Martin CK, Dawson JA (2017). Extended calorie restriction suppresses overall and specific food cravings: a systematic review and a meta-analysis. Obes Rev.

[53] Oustric P, Gibbons C, Beaulieu K, Blundell J, Finlayson G (2018). Changes in food reward during weight management interventions – a systematic review. Obes Rev.

[54] Anguah KO-B, Syed-Abdul MM, Hu Q, Jacome-Sosa M, Heimowitz C, Cox V, Parks EJ (2019). Changes in food cravings and eating behavior after a dietary carbohydrate restriction intervention trial. Nutrients..

[55] Apolzan JW, Myers CA, Champagne CM, Beyl RA, Raynor HA, Anton SA (2017). Frequency of consuming foods predicts changes in cravings for those foods during weight loss: the POUNDS Lost study. Obesity.

[56] Castro AI, Gomez-Arbelaez D, Crujeiras AB, Granero R, Aguera Z, Jimenez-Murcia S, Sajoux I, Lopez-Jaramillo P, Fernandez-Aranda F, Casanueva F (2018). Effect of a very low-calorie ketogenic diet on food and alcohol cravings, physical and sexual activity, sleep disturbances, and quality of life in obese patients. Nutrients..

[57] Chao AM, Wadden TA, Tronieri JS, Pearl RL, Alamuddin N, Bakizada ZM, Pinkasavage E, Leonard SM, Alfaris N, Berkowitz RI (2019). Effects of addictive-like eating behaviors on weight loss with behavioral obesity treatment. J Behav Med.

[58] Dorling JL, Bhapkar M, Das SK, Racette SB, Apolzan JW, Fearnbach SN, Redman LM, Myers CA, Stewart TM, Martin CK, CALERIE Study Group (2019). Change in self-efficacy, eating behaviors and food cravings during two years of calorie restriction in humans without obesity. Appetite..

[59] Drapeau V, Jacob R, Panahi S, Tremblay A (2019). Effect of energy restriction on eating behavior traits and psychobehavioral factors in the low satiety phenotype. Nutrients..

[60] Morin I, Bégin C, Maltais-Giguère J, Bédard A, Tchernof A, Lemieux S (2018). Impact of experimentally induced cognitive dietary restraint on eating behavior traits, appetite sensations, and markers of stress during energy restriction in overweight/obese women. J Obes.

[61] Smithson EF, Hill AJ (2017). It is not how much you crave but what you do with it that counts: behavioural responses to food craving during weight management. Eur J Clin Nutr.

[62] Watson NA, Dyer KA, Buckley JD, Brinkworth GD, Coates AM, Parfitt G, Howe PRC, Noakes M, Murphy KJ (2018). Reductions in food cravings are similar with low-fat weight loss diets differing in protein and carbohydrate in overweight and obese adults with type 2 diabetes: a randomized clinical trial. Nutr Res.

[63] Hill AJ, Brownell KD, Gold MS (2012). The psychology of food cravings. Food and addiction.

[64] Kettaneh A, Eclache V, Fain O, Sontag C, Uzan M, Carbillon L, Stirnemann J, Thomas M (2005). Pica and food craving in patients with iron-deficiency anemia: a case-control study in France. Am J Med.

[65] Louw VJ, du Preez P, Malan A, van Deventer L, van Wyk D, Joubert G (2007). Pica and food craving in adult patients with iron deficiency in Bloemfontein, South Africa. S Afr Med J.

[66] Moreno-Domínguez S, Rodríguez-Ruiz S, Fernández-Santaella MC, Ortega-Roldán B, Cepeda-Benito A (2012). Impact of fasting on food craving, mood and consumption in bulimia nervosa and healthy women participants. Eur Eat Disord Rev.

[67] Overduin J, Jansen A (1996). Food cue reactivity in fasting and non-fasting subjects. Eur Eat Disord Rev.

